# Responses to extreme drought in wintering waterbirds: a multi-species approach

**DOI:** 10.1186/s12983-025-00557-3

**Published:** 2025-02-10

**Authors:** Chenxi Wang, Shaoxia Xia, Xiubo Yu, Li Wen

**Affiliations:** 1 Key Laboratory of Ecosystem Network Observation and Modeling, Institute of Geographic Sciences and Natural Resources Research, Chinese Academy of Sciences, Beijing, 100101 China; 2 Science and Insights, Department of Climate Change, Energy, the Environment and Water, Parramatta, NSW 2150 Australia; 3 College of Resources and Environment, University of Chinese Academy of Sciences, Beijing, 100190 China

**Keywords:** Wintering waterbirds, Extreme weather events, Behavioral plasticity, Satellite tracking data, Home range

## Abstract

**Background:**

Climate change and anthropogenic activities are accelerating environmental changes, challenging wild animals’ survival. Behavioral plasticity, such as adjusting habitat selection and foraging activity, is a key mechanism for responding to rapid environmental changes in the Anthropocene era. However, this shift may expose animals to new challenges. Moreover, not all behavioral plasticity is adaptive, as evidenced by ecological traps. This study focuses on Poyang Lake, a Ramsar wetland and a critical wintering ground for waterbirds in the East Asian–Australasian Flyway. Historically, the migratory patterns of waterbirds were synchronized with the plant life cycle. However, recent hydrological regime changes have diminished suitable habitats and food resources, thereby posing significant conservation challenges for waterbirds.

**Methods:**

Utilizing multiyear satellite tracking data, we examined the variations in wintering home range and behaviors of four herbivorous waterbird species between natural and artificial wetlands in Poyang Lake under different hydrological conditions.

**Results:**

Our results reveal significant differences in home range area and movement speed among species and across hydrological years. All species demonstrated a marked increase in their use of artificial wetlands under unfavorable conditions. Specifically, the Greater White-fronted Goose (*Anser albifrons*) shifted its distribution to artificial wetlands during drought years while favoring natural wetlands under normal conditions, indicating a stress-induced adaptation. In contrast, the Bean Goose (*A. fabalis*) and Swan Goose (*A. cygnoid*) displayed greater behavioral plasticity. Notably, the Siberian Crane (*Leucogeranus leucogeranus*) increasingly used artificial wetlands, likely due to human protection, raising concerns about potential ecological traps. Additionally, waterbirds foraging in artificial wetlands generally exhibited higher movement speeds during drought conditions. This behavior suggests maladaptation and a more dispersed distribution.

**Conclusions:**

Our study underscored the critical role of artificial wetlands in supporting migratory waterbirds during drought, though elevated movement speeds observed in these habitats suggest potential maladaptation. Species-specific responses raise concerns about ecological traps if these habitats fail to meet key ecological needs. To ensure long-term conservation, efforts should focus on preserving natural wetlands and enhancing the quality of artificial habitats. Future research should prioritize long-term monitoring to guide habitat management and address species-specific needs in the face of climate change and habitat degradation.

**Graphic abstract:**

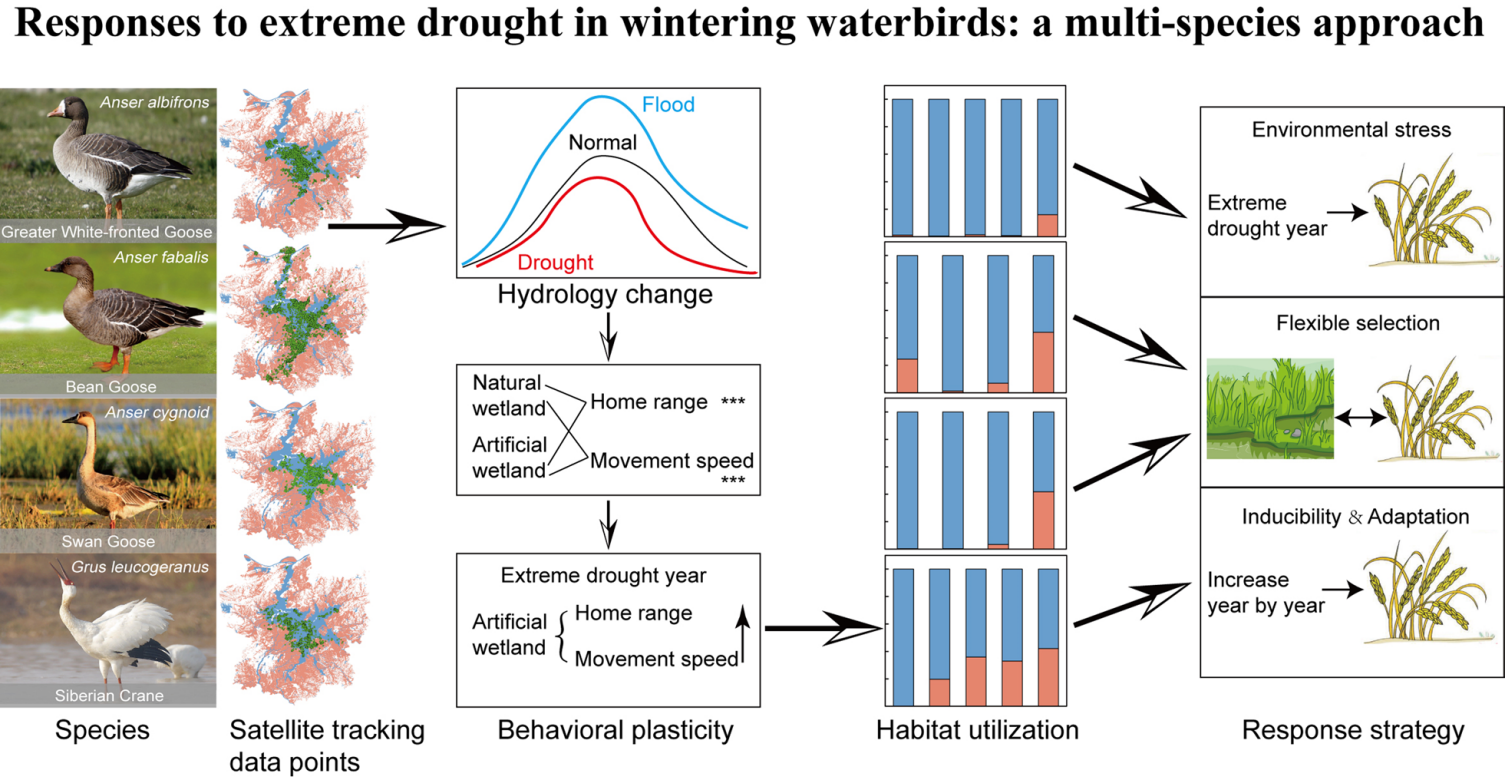

**Supplementary Information:**

The online version contains supplementary material available at 10.1186/s12983-025-00557-3.

## Article highlights

- Waterbirds increased utilization intensity and home range in artificial wetlands during unfavorable conditions, showcasing their behavioral plasticity in response to environmental changes.
- During droughts, waterbirds showed significantly increased movement speeds in artificial wetlands, indicating their maladaptation to these environments.
- Different species adopt various strategies to cope with the adverse effects of hydrological alternations.
- Wintering waterbird conservation requires species-specific management strategy.

**Supplementary Information:**

The online version contains supplementary material available at 10.1186/s12983-025-00557-3.

## Background

One way to cope with rapid environmental changes in the Anthropocene [1] is for animals to exhibit behavioral plasticity, such as modifying their dietary patterns [2, 3], habitat preferences [4] and other behavioral shifts [5]. This adaptability is particularly critical for wildlife in wetlands, which are increasingly threatened by climate change, population expansion, resource extraction, land reclamation, pollution, and invasive species [6, 7]. Waterbirds are key components of wetland ecosystems and serve as important indicators of their environmental quality and health [8]. Their high mobility enables them to exhibit remarkable behavioral flexibility in response to changing environmental conditions—a critical factor for their survival and reproductive success in dynamic ecosystems [9, 10].

Food resource availability is a key factor influencing habitat selection in waterbirds [11, 12]. According to the Optimal Foraging Theory, animals minimize time and energy costs while maximizing energy intake during foraging [13, 14]. When resources are abundant, waterbirds select nutrient-rich and easily accessible foods. During resource scarcity period, they may opt for lower-quality alternatives. Additionally, waterbirds choose relatively safe areas for foraging to minimize risks, such as predation and human disturbances [15]. In response to environmental changes, differences in individual behavioral plasticity led to varied survival strategies. For instance, some waterbirds migrate to cope with food shortages and environmental degradation, while others adapt by altering foraging behaviors and diets [2, 16]. This diversity in behavioral plasticity enables waterbirds to exhibit a high degree of adaptability under environmental stress, maintaining population stability and ecosystem integrity [17, 18].

Extreme hydrological events driven by climate change and extensive flow regulation have profound impacts on floodplain wetland ecosystems [19–21]. Under the combined effects of climate change and the operation of the Three Gorges Reservoir, significant hydrological alterations have occurred in Poyang Lake floodplain wetlands, a Ramsar site crucial for wintering waterbirds in the East Asian–Australasian Flyway (EAAF) [22, 23]. These changes include lowered dry season water levels and prolonged dry periods, which have become the new norm [24–26]. Additionally, frequent extreme hydrological events, such as the severe flooding in the summer of 2020 [27] and the extreme drought caused by prolonged high temperatures and low rainfall in 2022 [28], have led to the gradual degradation of habitats in Poyang Lake wetlands. These changes negatively affect the living conditions of the waterbirds, leading to habitat fragmentation and reduced food resources [29, 30]. However, the behavioral responses of waterbirds to these extreme hydrological events and the strategies they adopt remain unclear.

In North America, Europe, and part of Asia including Japan and South Korea, artificial wetlands such as farm dams, salt ponds, and rice paddies have become crucial habitats for waterbirds like cranes and geese [31–33]. However, in China, waterbirds still predominantly rely on natural wetlands [34]. With the ongoing changes in natural wetlands, some waterbirds may turn to artificial wetlands as alternative habitats [35]. At the same time, they may also adapt to new environments by altering their diets [36–38]. Despite this potential shift, the utilization of artificial wetlands by waterbirds in China have received relatively little attention, and conservation management practices for waterbirds in artificial wetlands are still in the early stages [39, 40].

This study investigates the habitat utilization characteristics and spatial shifts of four migratory waterbird species—Greater White-fronted Goose (*Anser albifrons*), Bean Goose (*A. fabalis*), Swan Goose (*A. cygnoid*), and Siberian Crane (*Leucogeranus leucogeranus*)—in Poyang Lake under varying hydrological conditions. Specifically, we investigate how these herbivores adjust their habitat use intensity between artificial and natural wetlands under unfavorable conditions, specifically those caused by extreme hydrological events that reduce the availability of suitable food resources and habitat area, utilizing multiyear (2018–2023) satellite tracking data. We further compare foraging behaviors between artificial and natural wetlands to explore the ecological and evolutionary consequences of these adaptations. By highlighting the importance of behavioral plasticity, we aim to contribute to a more comprehensive understanding of waterbird ecology and promote effective conservation efforts in the face of ongoing environmental change.

## Methods

### Study area

Poyang Lake, recognized as a Ramsar Wetland of International Importance, is one of the last two Yangtze-connected lakes, characterized by typical floodplain wetland features of great dynamics [41]. Its hydrology is driven by the prevailing sub-tropical monsoon climate. During the high-water period (June to September), the lake’s surface area exceeds 4125 km^2^, but it shrinks to just 500 km^2^ during the low-water period (November to March), when the water level can drop by 8–10 m. Specifically, the average water level of Poyang Lake during the dry season is 9.42 m (2017–2019, the same below), while during the wet season it is 18.14 m. The average water level during the rising water period is 12.42 m, and during the falling water period it is 13.45 m [42]. With water level withdrawal, numerous shallow sub-lakes emerge and the exposed lakebed is rapidly colonized by sedge plants such as *Carex* spp. This dynamic hydrology shapes diverse wetland habitats and abundant food resources [43], providing irreplaceable wintering grounds for many migratory waterbirds along the EAAF [44, 45]. Additionally, the water quality of Poyang Lake is relatively good, classified as mildly eutrophic, with no adverse effects on the growth of sandbar vegetation [46]. Notably, about 98% of the global Siberian Crane population [47], about 90% of the inland population of Swan Goose [48], about 70% of the East Asian continental population of the Greater White-fronted Goose [45], and about 50% of the East Asian population of the Bean Goose [49] overwinter here, demonstrating its strategic role in maintaining the stability of these migratory populations [50].

**Fig. 1:**
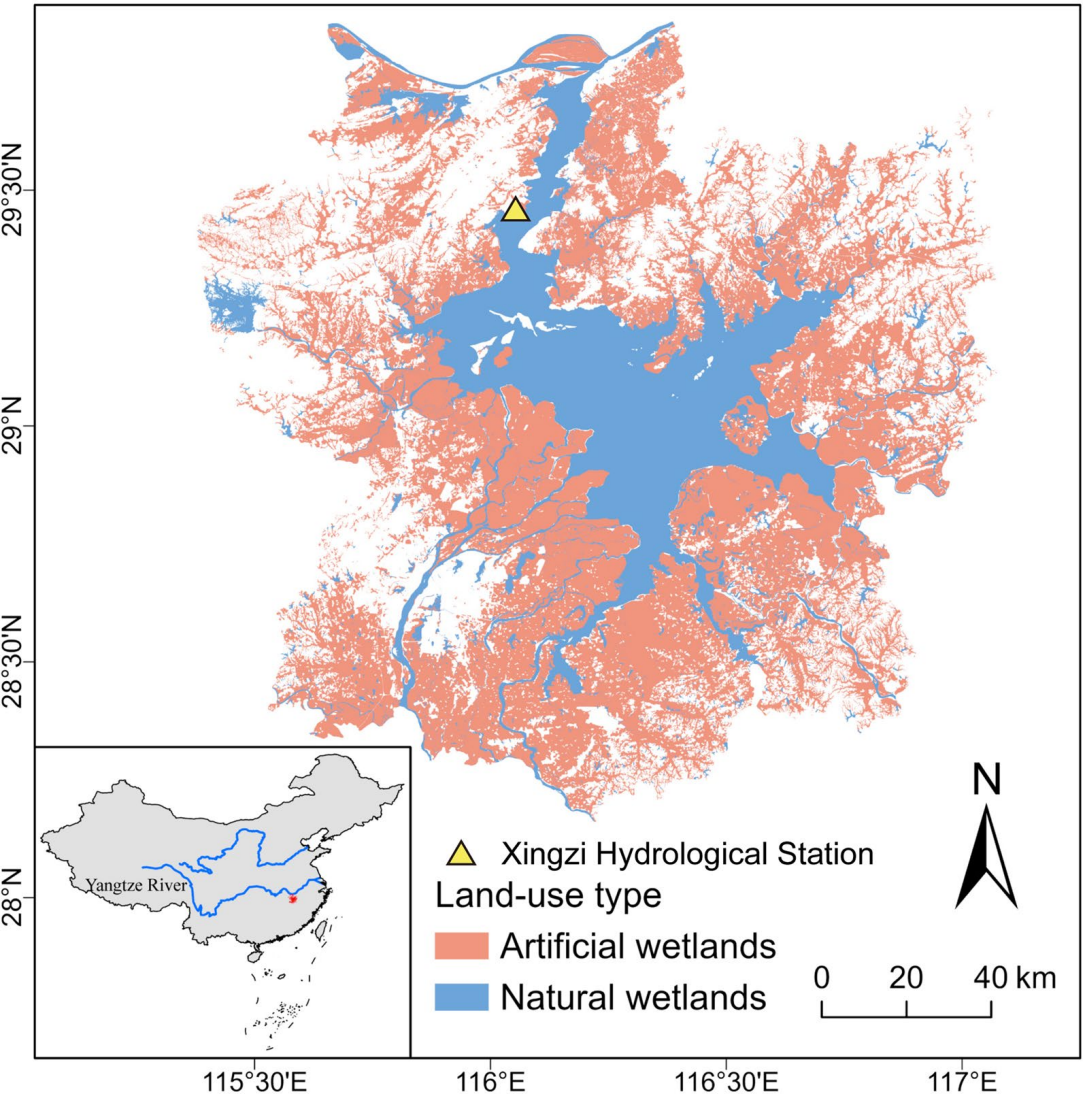
Map of the Study area and the distribution of natural and artificial wetlands. *Note*: Inset map shows the spatial relation between Poyang Lake and Yangtze River

The Poyang Lake Plain is also a vital grain (mainly rice) and aquaculture production (freshwater fish and shrimp) area in China. Rice paddies and aquaculture ponds have become potential habitats for wintering waterbirds during unfavorable years [37, 51]. To analyze waterbird utilization of natural and artificial wetlands, our study area (Fig. 1) includes the natural wetlands of Poyang Lake and the adjacent lowland agricultural areas. The artificial wetlands involved in this study include agricultural areas around Poyang Lake, such as farmlands, rice paddies, lotus ponds, and aquaculture ponds. The extent and area of these regions have remained stable throughout the study period, consisting of long-established and fixed land-use types.

We collected daily water level data from Xingzi Hydrological Station for 2018–2022 and extracted Poyang Lake’s surface area using the Google Earth Engine (GEE) platform [52]. Xingzi Hydrological Station is located on the left bank of Poyang Lake’s watercourse after the confluence of the five major rivers. Based on water level and surface area data during the recession period (October to December), we categorized the waterbird wintering periods (typically from October to March of the following year) into different hydrological years (Table 1).

**Table 1:** Water levels and surface areas (Mean ± SD) of Poyang Lake during the recession period (October-December)

Wintering period	Water level (m)	Water area (km^2^)	Type
2018–2019	10.78 ± 0.78	1601.85 ± 250.93	Normal year
2019–2020	8.91 ± 1.16	1201.71 ± 64.58	Normal year
2020–2021	12.79 ± 3.37	2551.36 ± 704.70	Flood year
2021–2022	11.24 ± 2.94	1874.54 ± 714.83	Normal year
2022–2023	7.13 ± 0.57	932.36 ± 129.61	Drought year

### Study species and their ecological traits

In this study, we selected four dominant species wintering in Poyang Lake, including the Greater White-fronted Goose, Bean Goose, Swan Goose, and Siberian Crane as focal species. The Greater White-fronted Goose and Bean Goose forage on grass stems and leaves, whereas the Swan Goose and Siberian Crane feed mainly on plant tuber and roots (Table 2).

**Table 2:** Food sources and preferred habitat of the target species [30, 36, 53–55]

Species	Feeding habits	Suitable habitat
*A. albifrons*	Newly emerged, low-height (120–220 mm) Poaceae and Cyperaceae (e.g., *Carex cinerascens* and *Phalaris arundinacea*) on sparse sandbars Also includes *Alopecurus aequalis*, *Cynodon dactylon*, and *Eleocharis migoana*	Sparse grasslands and mudflats, also in agricultural fields
*A. fabalis*		
*A. cygnoid*	Overwinter buds or tubers of submerged vegetation such as *Vallisneria spiralis* or *Potamogeton malaianus*. Grass-like plants (*Carex*) for Swan Goose, and tuberous roots of *Potentilla limprichtii* for Siberian Cranes	Shallow waters with < 40 cm depth or moist mudflats. Occasionally in marshes or croplands
*L. leucogeranus*		Shallow waters with < 50 cm depth or moist mudflats. Occurs in agricultural fields or lotus ponds

### Wetland classification and distribution

The land use data utilized in this study were sourced from the Resource and Environment Science Data Center (https://www.resdc.cn/). These data were primarily derived from 2020 Landsat OLI satellite imagery and interpreted through manual visual inspection, with a spatial resolution of 30 × 30 m. Wetland areas within the study region encompassed land use types such as cropland, lakes, reservoirs, ponds, and shoals (Table S1). Considering the habitat utilization patterns of waterbirds, wetland types were categorized into two classes: natural wetlands, consisting of lakes and shoals, and artificial wetlands, comprising cropland and reservoirs/ponds (Fig. 1).

### Satellite tracking data acquisition and analysis

#### Satellite tracking data acquisition and preprocessing

Satellite tracking technology provides high-frequency data on waterbird movement trajectories, offering essential support for accurately monitoring their behaviors and distribution dynamics [56, 57]. In 2018 and 2019, we conducted non-invasive captures of 5 Greater White-fronted Goose, 4 Swan Goose, 3 Siberian Crane, and 5 Bean Goose in Poyang Lake National Nature Reserve, Jiangxi, China. The birds were fitted with either neck-collar style (HQNG4625S, 30–45 g) or backpack-style (HQBG3621S, 24 g) satellite transmitters (Hunan Global Messenger Technology Co., Ltd.). Transmitter and harness attachments are less than 3% of body mass of the birds at the time of capture, ensuring that the deployment package weight remained within the acceptable body weight limits for birds (i.e., 3–5%) [58]. The solar-powered satellite transmitters, which included various sensors, returned data every hour, including the waterbird’s identification number, location (longitude, latitude), speed, heading, altitude, precision, and other information.

Individual satellite tracking data (Table 3) were exported in.xml format and categorized according to the wintering season. Data with precision levels A (5 m), B (10 m), and C (20 m) were selected based on accuracy criteria. The selected data were processed in ArcGIS to generate.shp format files, and extracted based on the study area boundaries [59] (Figure S1). Satellite transmitters return data every hour according to device settings, however, data loss due to weather or network issues occurred occasionally. To reconstruct waterbird movement trajectories, we employed the “*crawl*” package [60] in R 4.2.1 [61] to generate regular hourly waterbird trajectory data utilizing a space-state model [62].

**Table 3:** Information of satellite tracking data of waterbird

Species	No. of birds	Duration	Migration cycles	Number of fixes
*A. albifrons*	5	2018.10–2023.3	5	88,919
*A. fabalis*	5	2019.10–2023.3	4	197,171
*A. cygnoid*	4	2019.10–2023.3	4	103,460
*L. leucogeranus*	3	2018.10–2023.3	5	81,663

Each wintering period runs from October to March of the following year. Therefore, the specific time periods for the five wintering periods are as follows: 2018.10–2019.3, 2019.10–2020.3, 2020.10–2021.3, 2021.10–2022.3, and 2022.10–2023.3

#### Home range estimation and movement speed analysis

We used the dynamic Brownian Bridge Movement Model (dBBMM) from the R package “*move*” [63] to calculate the 95% home range for every bird during each wintering season (Figure S2). The home range areas in natural and artificial wetlands were extracted based on wetland type. Using the regularized waterbird trajectories generated in “Satellite tracking data acquisition and preprocessing” section, we applied the *as.ltraj* function from the R package “*adehabitatLT*” [64] to obtain the movement distance for each waterbird during each wintering season. The movement distances in natural and artificial wetlands were extracted separately for each bird, and movement speed was calculated based on these distances.

### Statistical analysis

We modelled habitat availability and use of different wetlands in relation to the hydrological conditions for each year and included these as categorical variables in our analysis. Specifically, for each type of year (flood, drought, normal), we have multiple individuals, ensuring that the data is not overly influenced by the unequal number of years. Since the repeated measurements are limited to a single wintering season for each individual, the risk of temporal correlation and dependency between measurements is reduced. Therefore, we believe that a two-way analysis of variance does not introduce significant bias or violate the assumption of independence. Specifically, we used the “*stats*” package in R to conduct a two-way analysis of variance (ANOVA) to examine the effects of different hydrological years and wetland types on waterbird home range area and movement speed, as well as potential interactions, assuming homogeneity of variance. If the data did not meet the homogeneity of variance assumption, to ensure homogeneity of variance, we applied a log transformation to the data. If the assumption was still not met, we employed non-parametric tests (Scheirer-Ray-Hare) using the “*rcompanion*” package [65] in R. Finally, if different hydrological years significantly affected waterbird home range area and movement speed, we performed post-hoc pairwise comparison tests (Duncan's Multiple Range test) using the “*agricolae*” package [66] to further investigate the differences in home range area and movement speed across different wetland types under various hydrological conditions.

## Results

### Species-specific home range variation

**Fig. 2:**
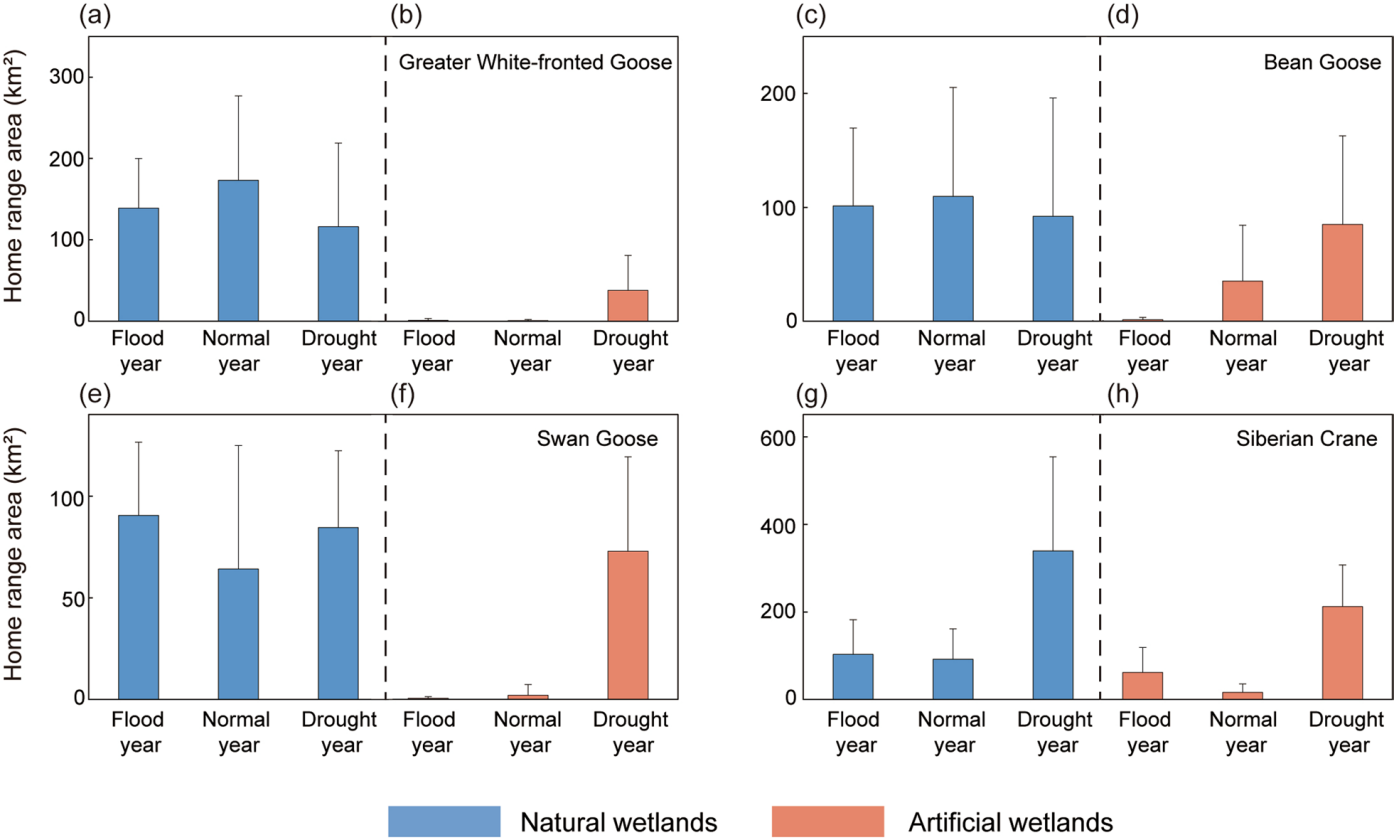
Home range areas of waterbirds in different wetland types during different hydrological years. Note the different scales of y-axis

**Fig. 3:**
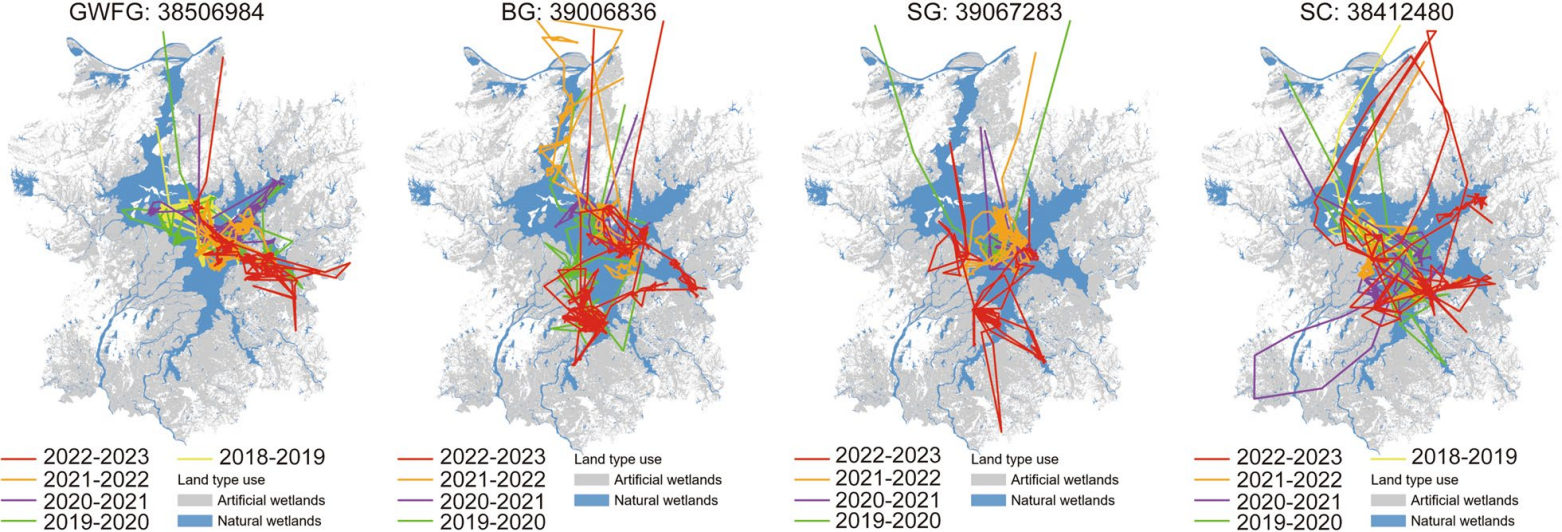
The movement trajectory maps of four waterbird species in different hydrological years

The Siberian Crane had the largest mean home range (Fig. 2g, h), followed by the Great White-fronted Goose (Fig. 2a, b) and Bean Goose (Fig. 2c, d) (which were comparable), and the estimated home range of Swan Goose was the smallest (Fig. 2e, f). The waterbirds exhibited different habitat utilization patterns across hydrological years, nevertheless, all species expanded their home range to into artificial wetlands during drought years (Figs. 2 and 3). Overall, the home range areas of Greater White-fronted Goose and Bean Goose did not show significant differences across different hydrological years (*p* > 0.05). However, wetland type had a significant effect on their home range areas (*p* < 0.05). For Swan Goose and Siberian Crane, both hydrological year and wetland type significantly affected their home range areas (*p* < 0.05) (Table 4). The post-hoc tested showed that there was no significant difference in home range area within natural wetlands across hydrological years (*p* > 0.05, Table 5) for the Swan Goose and Siberian Crane. However, their home range areas in artificial wetlands were significantly larger in drought year than in normal and wet years (*p* < 0.05 for all paired comparisons, Table 5). Notably, the home range areas of the Siberian Crane significantly increased in both natural and artificial wetlands during drought years. Additionally, the interaction between hydrological year and wetland type did not significantly affect the home range areas of any of the four waterbird species (*p* > 0.05) (Table 4).

**Table 4:** The effects of hydrological condition, wetland type, and their interaction on home range area

Species	Influencing factors	*df*	F	*p*
*A. albifrons*	Hydrological condition	2	0.17	0.92
	Wetland type	1	40.22	0.00***
	Hydrological condition × Wetland type	2	2.09	0.35
*A. fabalis*	Hydrological condition	2	2.21	0.33
	Wetland type	1	13.96	0.00***
	Hydrological condition × Wetland type	2	5.26	0.07
*A. cygnoid*	Hydrological condition	2	6.42	0.04*
	Wetland type	1	16.30	0.00***
	Hydrological condition × Wetland type	2	3.85	0.15
*L. leucogeranus*	Hydrological condition	2	7.54	0.02*
	Wetland type	1	4.54	0.03*
	Hydrological condition × Wetland type	2	1.07	0.59

Significant results are shown in bold. “×" represents an interaction. * represents p<0.05, ** represents p<0.01, *** represents p<0.001, all indicate a significant difference

**Table 5:** Post-hoc test comparing the difference in waterbird home range area across different hydrological conditions

Species		Influencing factors	Difference	S.E.	*p*
*A. cygnoid*	Natural wetland	Normal versus flood	− 26.35	29.85	0.39
		Normal versus drought	− 20.40	29.85	0.50
		Flood versus drought	5.95	34.47	0.87
	Artificial wetland	Normal versus flood	− 2.15	3.20	1.00
		Normal versus drought	− 10.45	3.20	0.01**
		Flood versus drought	− 8.30	3.69	0.01**
*L. leucogeranus*	Natural wetland	Normal versus flood	− 10.79	82.75	0.29
		Normal versus drought	− 247.00	96.63	0.29
		Flood versus drought	− 236.21	111.58	0.29
	Artificial wetland	Normal versus flood	− 45.93	37.52	0.25
		Normal versus drought	− 196.20	43.82	0.00***
		Flood versus drought	− 150.27	50.60	0.04*

Significant results are shown in bold. * represents p<0.05, ** represents p<0.01, *** represents p<0.001, all indicate a significant difference

### The proportion of home range in artificial wetlands

**Fig. 4:**
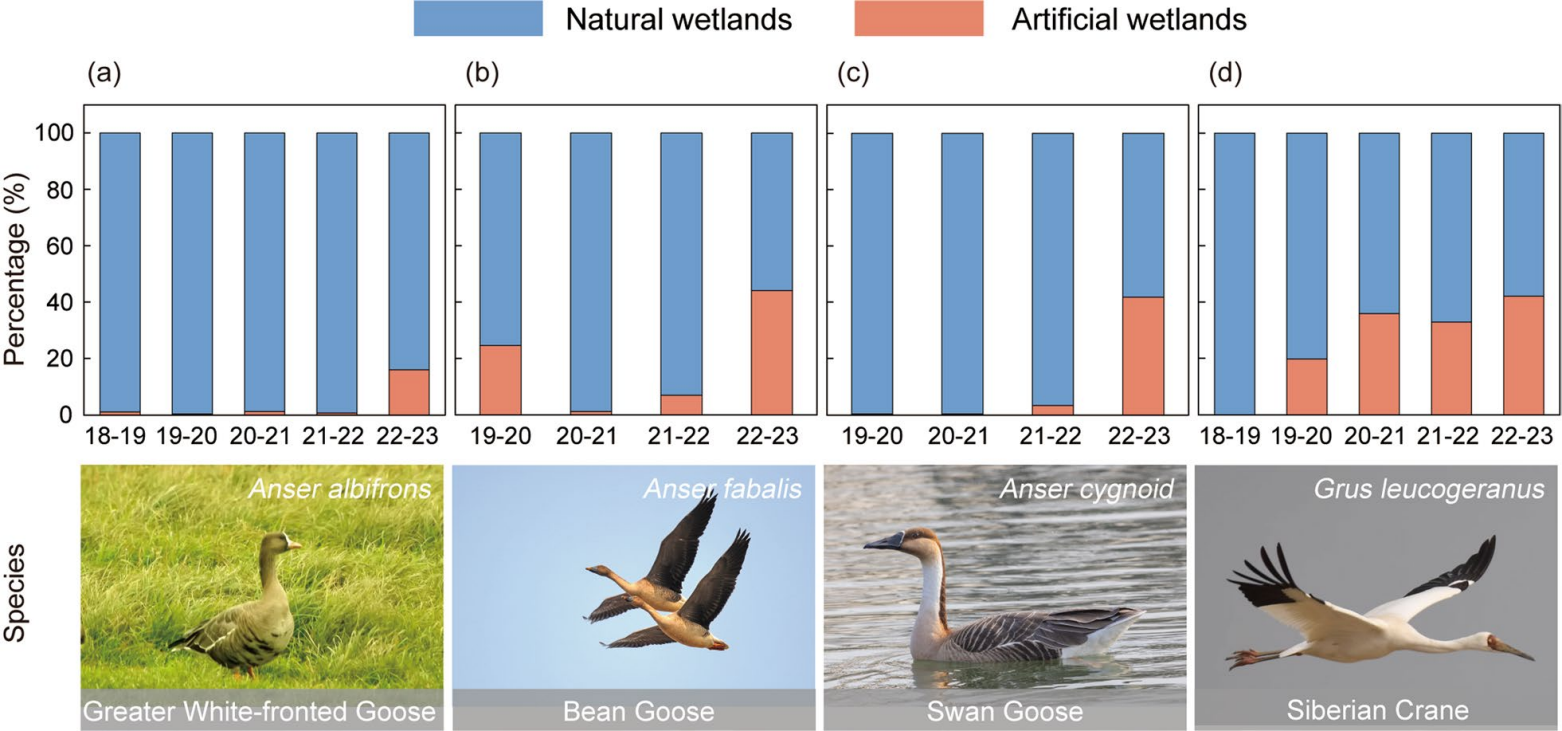
Distribution proportions of waterbirds in natural and artificial wetlands during different hydrological years. Greater White-fronted Goose, photographed by Yifei Jia. Bean Goose, photographed by Jianmin Wang. Swan Goose, photographed by Zhiquan Hao. Siberian Crane, photographed by Xiubo Yu

The proportion of home range within artificial wetlands varied across different hydrological years. The proportion of home range in artificial wetlands was highest in the 2022–23 wintering season, which was the driest year (Fig. 4). Specifically, the proportions for Greater White-fronted Goose, Bean Goose, Swan Goose, and Siberian Crane in artificial wetlands were 15.97%, 44.07%, 41.79%, and 42.09%, respectively (Fig. 4). The species exhibited different habitat utilization patterns in various hydrological years. The Greater White-fronted Goose used artificial wetlands only during drought years. In other years, it had very low utilization of artificial wetlands compared to other waterbirds, and stayed in primarily natural wetlands, which comprised over 99.00% of their distribution (Fig. 4a). On contrast, for Siberian Crane, the utilization of artificial wetlands increased annually since the begin of the study in the wintering season of 2019–2020 (Fig. 4d). Although the trends for Bean Goose and Swan Goose are less pronounced, their utilization of artificial wetlands has also increased (Fig. 4b, c).

### Movement speed variation across study species

**Fig. 5:**
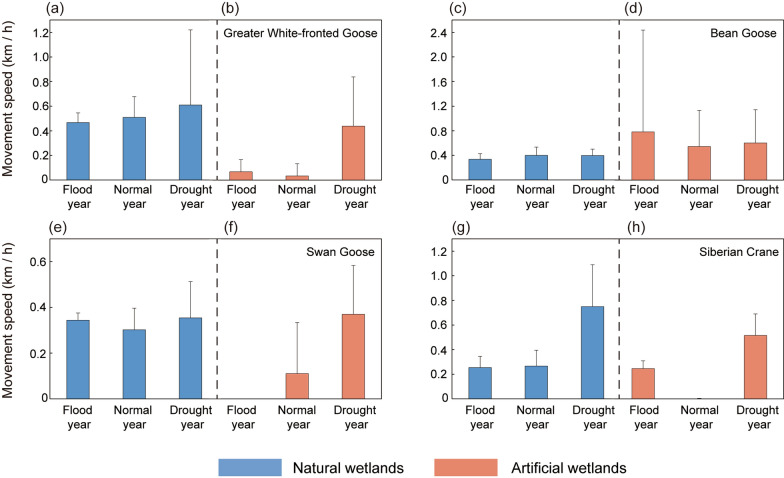
Movement speed of waterbirds in different wetland types during different hydrological years

Greater White-fronted Goose, Swan Goose, and Siberian Crane exhibited the highest movement speeds during extreme drought year, regardless of whether they were in natural or artificial wetlands (Fig. 5a, b, e–h). In contrast, Bean Goose showed no significant differences in movement speed across different hydrological years (*p* > 0.05) (Fig. 5c, d). Except for Bean Goose, the movement speeds of the other three species differed significantly across hydrological years and wetland types (*p* < 0.05) (Table 6). Specifically, the movement speed of the Greater White-fronted Goose, Swan Goose, and Siberian Crane did not change significantly in natural wetlands across wintering seasons (*p* > 0.05) (Table 7). However, in artificial wetlands, their movement speeds were significant greater in drought year than in normal years (*p* < 0.05) (Table 7). Additionally, for the Swan Goose, the movement speed in extreme drought year was significantly higher than in both normal years and extreme wet year in artificial wetlands. For the Siberian Crane, the movement speed in both extreme drought and extreme wet year was significantly higher than in normal years in artificial wetlands (*p* < 0.05) (Table 7). Additionally, the interaction between hydrological year and wetland type significantly affected the movement speed of Greater White-fronted Goose (*p* < 0.05), but had no impact on the movement speeds of Bean Goose, Swan Goose, or Siberian Crane (*p* > 0.05) (Table 6).

**Table 6:** The effects of hydrological condition, wetland type, and their interaction on waterbird movement speed

Species	Influencing factors	df	F	*p*
*A. albifrons*	Hydrological condition	2	2.58	0.01**
	Wetland type	1	31.22	0.00***
	Hydrological condition × wetland type	2	6.37	0.04*
*A. fabalis*	Hydrological condition	2	3.58	0.17
	Wetland type	1	0.14	0.71
	Hydrological condition × wetland type	2	0.79	0.67
*A. cygnoid*	Hydrological condition	2	6.12	0.03*
	Wetland type	1	7.49	0.01**
	Hydrological condition × wetland type	2	3.21	0.20
*L. leucogeranus*	Hydrological condition	2	9.71	0.01**
	Wetland type	1	6.58	0.01**
	Hydrological condition × wetland type	2	3.17	0.21

Significant results are shown in bold. “×” represents an interaction. * represents p<0.05, ** represents p<0.01, *** represents p<0.001, all indicate a significant difference

**Table 7:** Post hoc test comparing the difference of in waterbird movement speed across different hydrological conditions

Species		Influencing factors	Difference	S.E.	*p*
*A. albifrons*	Natural wetland	Normal versus wet	0.09	0.14	0.54
		Normal versus drought	− 0.17	0.14	0.24
		Wet versus drought	− 0.26	0.18	0.15
	Artificial wetland	Normal versus wet	− 0.04	0.10	0.69
		Normal versus drought	− 0.48	0.10	0.00***
		Wet versus drought	− 0.43	0.12	0.21
*A. cygnoid*	Natural wetland	Normal versus wet	− 0.04	0.06	0.51
		Normal versus drought	− 0.05	0.06	0.42
		Wet versus drought	− 0.01	0.07	0.89
	Artificial wetland	Normal versus wet	0.11	0.11	0.34
		Normal versus drought	− 0.26	0.11	0.04*
		Wet versus drought	− 0.37	0.13	0.01**
*L. leucogeranus*	Natural wetland	Normal versus wet	0.01	0.13	0.13
		Normal versus drought	− 0.48	0.16	0.13
		Wet versus drought	− 0.50	0.18	0.13
	Artificial wetland	Normal versus wet	− 0.25	0.06	0.02*
		Normal versus drought	− 0.51	0.07	0.01**
		Wet versus drought	− 0.27	0.08	1.00

Significant results are shown in bold. * represents p<0.05, ** represents p<0.01, *** represents p<0.001, all indicate a significant difference

## Discussion

The study of behavioral plasticity in waterbirds not only enhances our understanding of their ecology and evolution but also has significant implications for conservation [5, 67]. As climate change and human activities continue to modify aquatic ecosystems [68–70], the ability of waterbirds to adapt to these changes will be critical for their persistence [3, 71]. Moreover, understanding the mechanisms underlying behavioral plasticity can inform conservation strategies aimed at protecting and restoring habitats, ensuring that waterbirds have the necessary resources and conditions to exhibit adaptive behaviors. This study used multiyear satellite tracking data to investigate the habitat use intensity and behavioral variations in artificial and natural wetlands of four migratory species in a crucial wintering ground, and to explore their behavioral plasticity in response to accelerated environmental changes. By bridging the gap between ecological theory and practical conservation, the findings provide a vital foundation for formulating strategies to mitigate the impacts of global change on waterbirds and their habitats.

### Impact of hydrological changes on waterbird food resources

Global hydrological extremes, such as flooding and drought, have become more frequent in recent decades [72], with intensified river regulation further exacerbating these events [21, 73]. These changes have profound impacts on floodplain wetland ecosystems [19, 20], where hydrological regimes play a key role in shaping the distribution and growth of wetland plants [74, 75]. The resulting shifts in plant distribution directly affect the availability of food resources for waterbirds [76], which can lead to significant changes in wetland ecosystems and contribute to declines in waterbird populations [77].

The dynamic hydrological conditions of Poyang Lake create a diverse and heterogeneous habitat, providing high-quality food resources for wintering waterbirds [78]. Tuber-eating species, including geese and cranes, depend heavily on winter buds and tubers from submerged plants like *Vallisneria spiralis* and *Potamogeton malaianus* [54, 55]. However, recent declines in submerged plants, particularly *Vallisneria spiralis*, in the middle and lower reaches of the Yangtze River [49, 79] have been associated with declines in waterbird populations [80]. High-water levels and flooding events during the summer months can severely damage submerged plant habitats [81, 82], leading to shortages in food resources for these tuber-eating species [51, 83]. For instance, the summer flooding event of 2020 in Poyang Lake likely reduced the formation and growth of *Vallisneria spiralis* tubers, causing food shortages for wintering waterbirds during the 2020–2021 period [27, 84]. This forced waterbirds to expand their foraging efforts into artificial wetlands, such as lotus ponds and rice fields [36].

In contrast, droughts with low-water levels promote the invasion of emergent plants [85], which can restrict the availability of food resources for waterbirds. This limitation of food resources is one reason why Siberian Cranes and Swan Geese increase their reliance on artificial wetlands during both flooding and drought years (Fig. 2).

For herbivorous waterbirds, newly emerged low-growing plants like *Carex cinerascens* and *Phalaris arundinacea* serve as essential food sources [30, 53]. Hydrological extremes disrupt the synchronization between plant growth and waterbird migration, causing phenological mismatches that hinder waterbird access to suitable food [86]. During the 2022–2023 wintering season, earlier lakebed exposure led to overgrowth of *Carex* spp., causing a shortage of high-quality food in natural wetlands. This may be the reason why there is an increased distribution of geese in artificial wetlands (Figs. 2 and 3). In contrast, excessive water levels in wet years reduced the area of *Carex* wet meadows, limiting foraging opportunities and making it more difficult for them to access suitable food resources, though this effect was most pronounced in the early wintering period [77, 87].

### Waterbirds’ response strategy to hydrological extreme events

The selection of food resources by waterbirds is in line with Optimal Foraging Theory, which suggests that animals prioritize food sources that offer the highest nutritional returns [13, 14]. In Poyang Lake, species such as the Greater White-fronted Goose and Bean Goose prefer *Carex* spp., while the Siberian Crane and Swan Goose favor Vallisneria tubers [30, 51]. These food resources are primarily found in natural wetlands, including shallow water areas, mudflats, and wet meadows, accounting for the higher prevalence of waterbirds in these habitats. When food resources become scarce due to extreme hydrological events, waterbirds exhibit dietary shifts. For example, Siberian Cranes may feed on *Potentilla limprichtii* and lotus roots (Nelumbo) [36, 51], while Swan Geese turn to *Carex* [37]. Additionally, waterbirds adapt to harsh conditions by expanding their foraging range [3].

In selecting habitats, waterbirds weigh food availability against the risk of predation and other dangers [88, 89]. Natural wetlands, characterized by relatively low human presence, are typically preferred foraging sites [39, 40]. In China, wintering waterbirds primarily use natural wetlands, as cultivated agricultural areas do not offer safe foraging opportunities [34]. However, artificial wetlands, though often used more frequently during drought periods, are associated with maladaptive behaviors. Increased movement speeds observed in these artificial wetlands (Fig. 4) suggest that waterbirds are not fully adapted to these environments. Artificial wetlands in Poyang Lake are fragmented and often require waterbirds to fly long distances in search of suitable food patches, leading to higher movement speeds [40]. Furthermore, artificial wetlands experience higher levels of human disturbance, including bird scaring and domestic animals, contributing to increased flight responses [90]. As a result, the increased use of artificial wetlands during drought years represents not an optimal strategy but a temporal response to food scarcity.

### Maladaptation and ecological trap risk

The reliance on artificial wetlands by species such as Siberian Cranes and Swan Geese during periods of drought raises concerns about the potential for ecological traps [91–93]. These artificial wetlands provide immediate food resources, but they may also expose waterbirds to significant risks, such as increased predation pressure, human disturbance, and disease outbreaks due to congregation in high-density areas [94]. The growing preference for artificial wetlands, particularly by the Siberian Crane, may be driven by continuous food supplementation, which has made these habitats more attractive compared to natural wetlands [95, 96]. However, this shift in habitat use could have long-term consequences for the species, particularly if artificial wetlands continue to attract large numbers of waterbirds, potentially leading to maladaptive behaviors and population-level effects.

The risk of disease outbreaks in artificial wetlands, where waterbird densities are high, is another concern. Increased congregation in these habitats can facilitate the spread of pathogens, leading to greater vulnerability among waterbird populations [97]. Research into the ecological risks associated with artificial wetlands is crucial for understanding the long-term consequences of habitat shifts and the role of disease dynamics in these environments.

### Research limitations and the role of individual experience in habitat selection

While our study provides valuable insights into the changes in waterbird habitat use under extreme hydrological conditions, certain limitations should be acknowledged. Specifically, the small sample size and the limited environmental variability, encompassing only one drought year and one flood year, may restrict the generalizability of the results. Nevertheless, the extensive individual data collected over a prolonged period largely compensates for these constraints and provides a robust foundation for understanding habitat use during hydrological extremes. Additionally, time-of-day effects (e.g., day/night differences) could further enhance the analysis of habitat use, but this was not the focus of the current study. Exploring this aspect could be a valuable direction for future research.

Furthermore, individual experience plays a crucial role in habitat selection, influencing how waterbirds respond to environmental changes. As birds age, they accumulate experience, which may improve their ability to find food, avoid predators, and identify suitable habitats. Older individuals often exhibit more efficient foraging strategies and are better at navigating environmental challenges [98, 99]. This experience could influence how waterbirds select habitats, especially during periods of environmental stress, such as hydrological extremes. However, the influence of individual experience on habitat selection in response to environmental stressors remains understudied. Future research is needed to explore how prior exposure to different habitat types and environmental conditions affects waterbird decision-making. Experimental studies that manipulate exposure to various habitat conditions and track the behavior of individuals could provide valuable insights into how experience shapes habitat selection, particularly in the context of changing environmental conditions.

## Insights for wetland and waterbird conservation and conclusion marks

### Management of artificial wetland as an important alternative habitat

The behavioral plasticity demonstrated in this study suggests that artificial wetlands serve as important alternative habitats during hydrological extreme events. These wetlands are highly fragmented, small in size, and subject to significant human interference. Consequently, waterbirds need to invest more time searching for food and remain vigilant due to human disturbances (Table 6). Management measures for farmlands in the Poyang Lake area could draw lessons from practices in Europe, South Korea, and Japan [35, 100]. In addition to controlling water levels in rice fields and leaving sufficient food after harvesting [101], it is particularly recommended to establish mechanisms for ecological compensation for farmers to prevent human-bird conflicts. This includes reducing threats to waterbird safety, such as domestic animals and activities like herding and bird trapping [90, 102–105].

Furthermore, due to species-specific adaptation strategies, community-based conservation is essential. The formulation of conservation policies needs to consider the adaptability of waterbirds and the risk levels of alternative habitats, shifting from existing strategies focused on individual species to a comprehensive consideration of the needs of different species. Our study indicates that the Siberian Crane, the flagship species in Poyang Lake, garners high international and national attention [106] and holds a high conservation status. Existing artificial habitat creation and supplemental food supply might sufficiently mitigate the impact of extreme hydrological events on this species [51]. However, continuous food supplementation is not encouraged to avoid ecological traps [92]. Conversely, the Bean Goose and the Swan Goose, with relatively diverse food resource preferences and flexible behavioral plasticity, demonstrate stronger adaptability [37]. The Greater White-fronted Goose, however, shows weaker adaptability and relies more on natural wetlands. Despite this, due to its lower conservation status and less public attention, frequent extreme hydrological events may pose a high risk of rapid population decline. Therefore, it is crucial to protect the remaining natural wetlands for the Greater White-fronted Goose [107], as these habitats are vital for its survival.

Therefore, in formulating conservation strategies, it is essential to fully consider species-specific needs and differences in adaptability to extreme climates and environmental changes. Comprehensive conservation management should be based on the specific needs of different waterbird species, strengthening monitoring of the population size and breeding status of the Greater White-fronted Goose, and adjusting its conservation status accordingly. In conclusion, adopting a comprehensive approach involving natural wetland restoration, hydrological management, artificial wetland protection, and the formulation of integrated conservation strategies is essential for maintaining the stability and diversity of waterbird populations [107].

### Conservation of natural wetlands as management priority

While the use of artificial wetlands increased during hydrological extreme events, particularly droughts, natural wetlands remained the primary wintering habitat for the studied species (Figs. 2 and 3). The irreplaceable nature of these wetlands underscores the importance of their restoration. Various habitat conservation actions could be implemented to maintain the ecological integrity of the Ramsar wetland.

The recent large-scale “Ten-Year Fishing Ban in the Yangtze River” has exacerbated the grazing pressure of herbivorous fish on submerged plants. This has led to a decline in these plants, which are crucial food sources for tuber-feeding waterbirds [108]. Furthermore, persistent low-water levels in recent years have disrupted the balance between the migration patterns of wintering geese and the growth cycles of their food resources. This has resulted in the premature aging of *Carex*, significantly reducing the availability of food for herbivorous waterbirds [109]. To address these issues, restoration of food resources in natural wetlands could include actions such as replanting submerged vegetation [51] and mowing overmatured wet meadows to regulate the growth cycles of *Carex* [110]. In the case of Poyang Lake, where mild eutrophication and favorable water quality prevail [28], planting submerged vegetation thus presents a particularly promising solution for restoration. These actions would improve both the quantity and dietary quality of essential food resources for waterbirds. Natural sub-lakes, the main distribution areas for waterbirds, have achieved a win–win situation for both fisheries resources and waterbird conservation through traditional fishing activities [111]. However, to further accommodate the ecological needs of vegetation growth and waterbird habitat, it is recommended to develop water level management plans for these sub-lakes. For instance, delaying the water level drawdown time of the sub-lakes can prevent premature aging of *Carex* [30, 53].

In summary, adopting a comprehensive approach involving natural wetland restoration, hydrological management, artificial wetland protection, and the formulation of integrated conservation strategies accommodating the species-specific behavioral plasticity and response strategy is essential for maintaining the stability and diversity of waterbird populations.

## Supplementary Information

Additional file1 (DOCX 18413 KB)

## Data Availability

The data supporting this article will be available upon request.
