# Supplementary material for: Responses to extreme drought in wintering waterbirds: a multi-species approach
Source: Front Zool. 2025 Feb 10;22:3. doi: 10.1186/s12983-025-00557-3 (PMC11809017; doi:10.1186/s12983-025-00557-3)
Supplement: Supplementary file 1 — Additional file1 (DOCX 18413 KB) [file 12983_2025_557_MOESM1_ESM.docx]

# Supplemental information


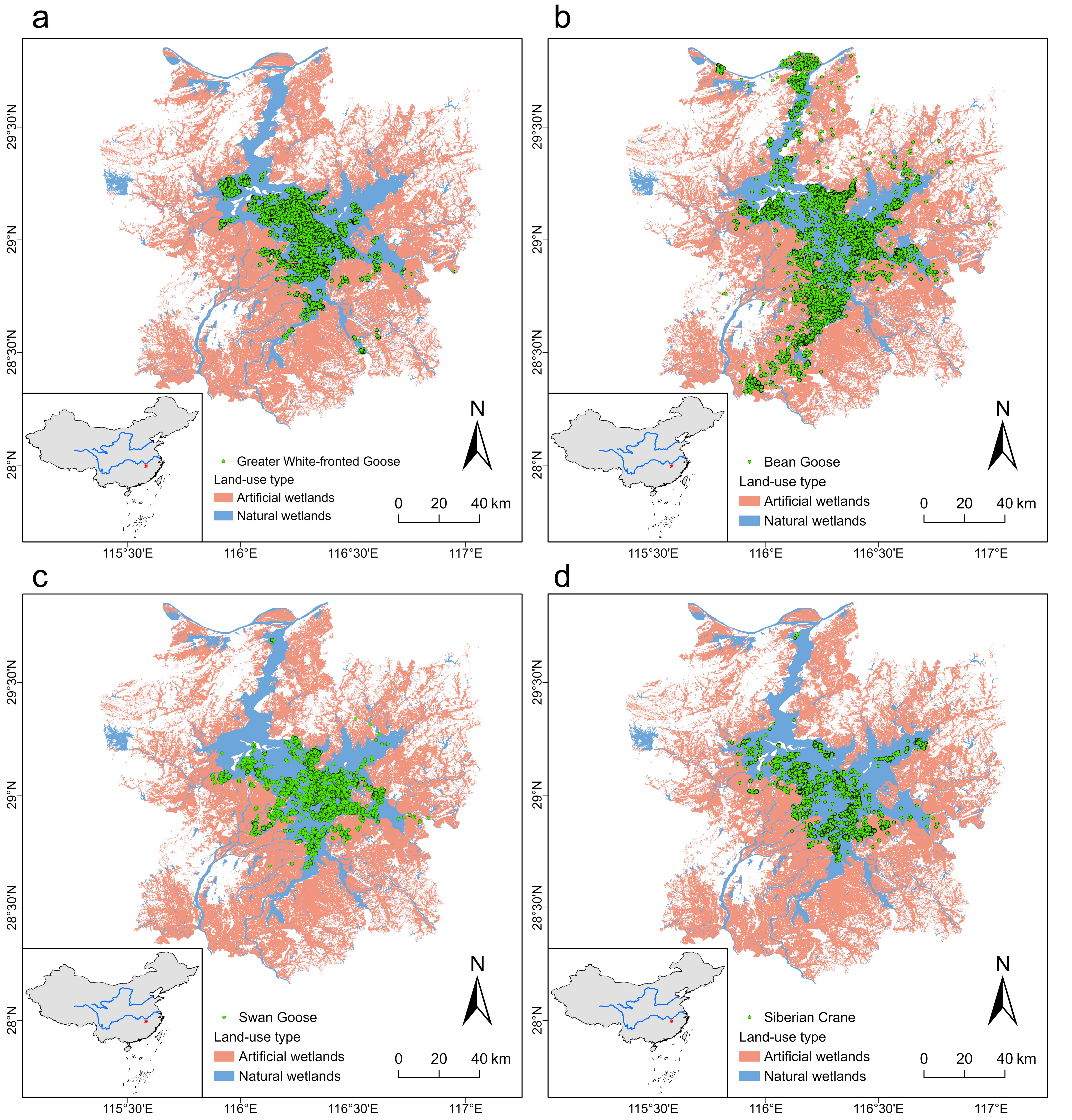


Figure S1. Different waterbird satellite tracking data point information


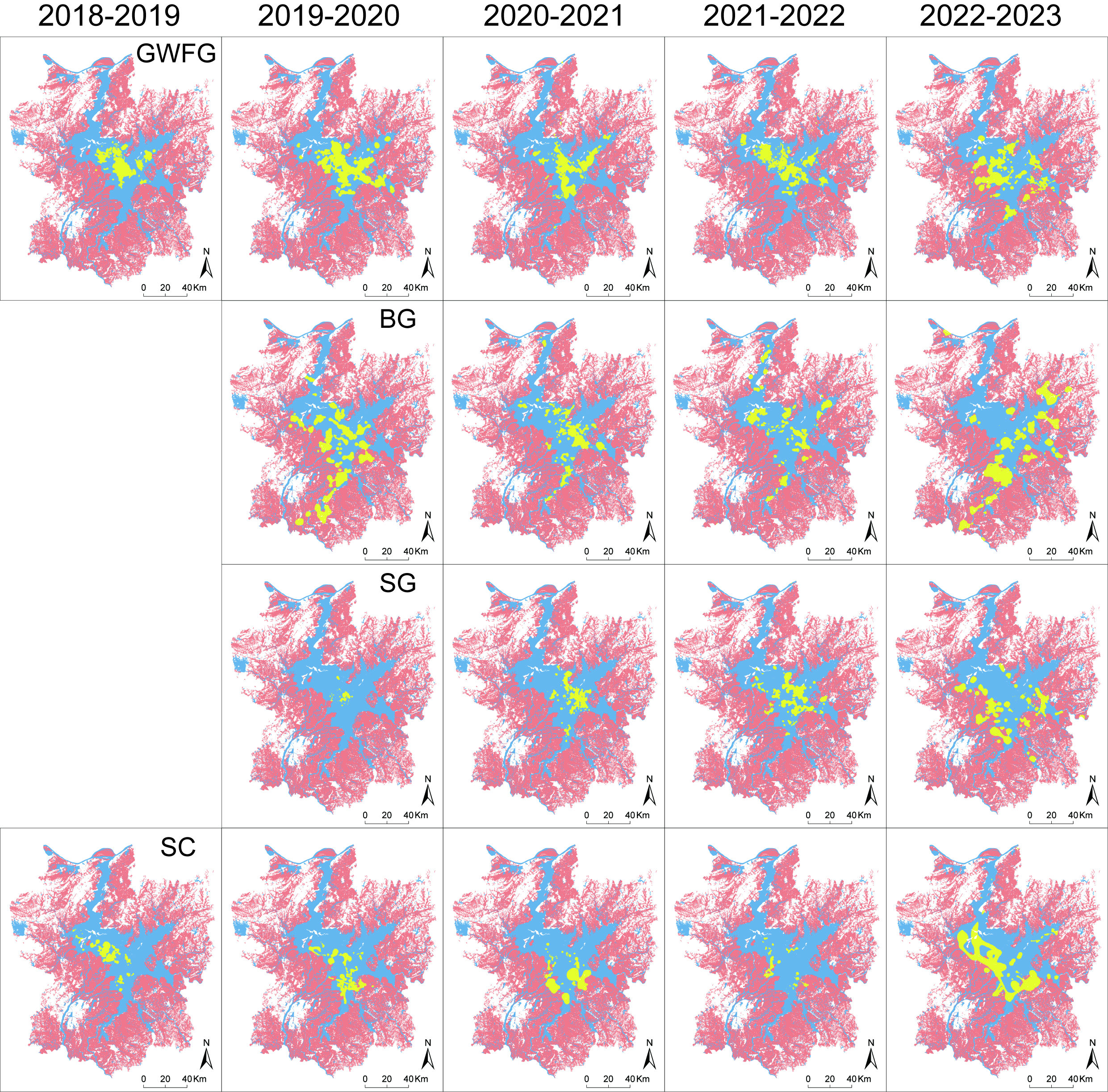


Figure S2. Different waterbird home range distributions in different hydrological years.

*Note*: GWFG represents the Greater White-fronted Goose, BG represents the Bean Goose, SG represents the Swan Goose, SC represents the Siberian Crane.

Table S1. Land use type classification

| **Number** | **Name** | **Definition** | **Remote sensing image feature** |
| --- | --- | --- | --- |
| 1 | Croplands | Land on which crops are grown, including paddy fields and drought lands | The features are geometrically distinct, with large and flat areas characterized by elongated and strip-like structures. The tones are uniform and smooth, lacking granularity. Drought land areas exhibit blue-cyan and white-green tones, while irrigated areas show shades of red, dark red, light red, and black-gray. The imagery appears as large patch-like formations. |
| 2 | Lakes and rivers | Refers to naturally formed waterlogged areas of land below the perennial water level | The geometric features are distinct, exhibiting natural curves or localized straight sections with easily delineated boundaries, and the image colors are extremely uniform. Lakes appear with deep blue or navy hues, while rivers exhibit light blue and white-green tones. |
| 3 | Reservoirs/ponds | Refers to the land below the perennial water level of the artificially constructed water storage area | The geometric shape is distinct with evident signs of human construction, featuring shades of deep blue, blue, and light blue in the imagery. The image texture is fine, with clear textures and uniform colors. |
| 4 | Shoals | It refers to the land between the water level of rivers and lakes in the normal period and the flood period | The imagery displays irregular stripes or patches, characterized by shades of gray, light gray, and white, with a fine texture and uniform color tones. |
